# Predictors and 3‐year outcomes of compromised left circumflex coronary artery after left main crossover stenting

**DOI:** 10.1002/clc.23693

**Published:** 2021-07-16

**Authors:** Hameed Ullah, Karim Elakabawi, Han Ke, Najeeb Ullah, Habib Ullah, Sardar Ali Shah, Hamad Haider Khan, Muhammad Asad Khan, Ning Guo, Zuyi Yuan

**Affiliations:** ^1^ Department of Cardiology First Affiliated Hospital of Xi'an Jiaotong University Xi'an China; ^2^ Department of Cardiology Benha University Egypt; ^3^ Department of Data Science (FIT) University of Monash Molbourne Australia; ^4^ Department of Cardiology Dow University of health sciences Karachi Pakistan; ^5^ Department of Endocrinology Xi'an Jiaotong University Xi'an China; ^6^ Department of Cardiology Hayatabad Medical Complex Peshawar Pakistan

**Keywords:** compromised left circumflex artery, drug‐eluting balloon, fraction flow reserve, intravascular ultrasound, left main stem, percutaneous interventions

## Abstract

**Background:**

There are few predictors of decreased fractional flow reserve (FFR) in the left circumflex coronary artery (LCx) after left main (LM) crossover stenting.

**Objectives:**

We aimed to determine the predictors for low FFR at LCx and possible treatment strategies for compromised LCx, together with their long‐term outcomes.

**Methods:**

Altogether, 563 patients who met the inclusion criteria were admitted to our hospital from February 2015 to November 2020 with significant distal LM bifurcation lesions. They underwent single‐stent crossover percutaneous coronary intervention (PCI) under intravascular ultrasound (IVUS) guidance with further LCx intervention based on the measured FFR.

**Results:**

The patients showed significant angiographic LCx ostial affection post‐LM stenting, but only 116 (20.6%) patients had FFR < 0.8. The three‐year composite major adverse cardiac events (MACE) rates were comparable between the high and low FFR groups (16.8% vs. 15.5; *p* = 0.744). In a multivariate analysis, low FFR at the LCx was associated with post‐stenting minimal luminal area (MLA) of LCx (odds ratio [OR]: 0.032, *p* < .001), post‐stenting LCx plaque burden (OR: 1.166, *p* < .001), poststenting LM MLA (OR: 0.821, *p* = .038), and prestenting LCx MLA (OR: 0.371, *p* = .044). In the low FFR group, those with compromised LCx managed with drug‐eluting balloon had the lowest three‐year MACE rate (8.1%), as compared to either those undergoing kissing balloon inflation (KBI) (17.5%) or stenting (20.5%) (*p* = 0.299).

**Conclusion:**

Unnecessary LCx interventions can be avoided with FFR‐guided LCx intervention. Poststenting MLA and plaque burden of the LCx, and main vessel stent length are poststenting predictors of low FFR.

## Highlights

- FFR‐guided LCx intervention avoided 79.4% unnecessary LCx intervention with comparable 3‐ year MACE rates between high FFR patients and patients who had low FFR and adequately managed.
- IVUS‐measured equivalent of low FFR in the LCx include Pre‐PCI LCx MLA and PostPCI LCx MLA, LCx plaque burden, and LM MLA.
- Treatment of compromised LCx with DEB carried the lowest 3‐year clinical adverse events.

## 1 INTRODUCTION

Despite considerable improvements in percutaneous coronary intervention (PCI) techniques and pharmacotherapeutics, distal left main (LM) coronary artery lesions continue to be one of the most difficult interventional cardiology objectives.^1^ The single‐stent strategy is superior to the two‐stent approach, and is regarded as the treatment of choice whenever feasible for LM bifurcation lesions,^2^ even though the predictors, functional significance, and luminal changes for compromised side‐branch ostium after main vessel (MV) stenting have a large degree of divergence.^3^ Previous studies have indicated that plaque or carina shift are the two main mechanisms for compromised left circumflex coronary artery (LCx) after LM to left anterior descending (LAD) artery stenting.^4^ Coronary angiography is the most used method to measure the extent of atherosclerotic disease and evaluate the geometric changes associated with stent implantation; however, the discordance between anatomic stenosis as evaluated by angiography or intravascular ultrasound (IVUS), and the functional significance of the jailed LCx is not well known.^5^, ^6^ Fractional flow reserve (FFR)‐guided LCx interventions have been proposed to improve clinical outcomes by reducing unnecessary procedures.^7^ FFR‐guided LCx intervention may be technically difficult, and its superiority over angiography‐guided provisional LCx intervention is still questionable.^8^, ^9^ Thus, we aimed to provide IVUS‐guided predictors for low FFR at LCx after LM to LAD crossover stenting, and to compare the different treatment strategies for compromised LCx and their long‐term outcomes.

## 2 METHODOLOGY

### 2.1 Study design

This single‐center retrospective study was conducted at the Department of Cardiology, First Affiliated Hospital of Xian Jiaotong University, PR China, from February 2015 to November 2020. We initially included 1974 consecutive patients with significant de novo LM stem coronary artery disease with angiographic stenosis (>50%), distal bifurcation lesions with Medina classification (1,1,0), and mild LCx disease of angiographic stenosis (<50%). Exclusion criteria were (1) patients who had received prior PCI or coronary artery bypass grafting (CABG); (2) patients with left ventricular ejection fraction (LVEF) < 35%; (3) LCx‐angiographic stenosis >50% on pre‐PCI coronary angiography; (4) diffuse LCx disease >7 mm from the LCx ostium; (5) patients with visible thrombi in the LCx territory; (6) patients with LCx‐angiographic diameter < 2.5 mm; (7) patients with acute myocardial infarction (MI) in any coronary artery; (8) patients with clear angiographic evidence of non‐comprised LCx postPCI; and (9) patients who had a failure in LCx re‐wiring or IVUS pull back from LCx to LM.

Finally, 563 patients, who showed a significant or possibly significant ostial LCx lesion as evidenced by angiographic diameter stenosis (DS > 50%) after MV stenting, were enrolled. Independent IVUS pullback from the LAD to LM and LCx to LM with minimal luminal area (MLA), minimal luminal diameter (MLD), and plaque burden (PB) measurements were performed at each epicardial coronary artery for the final analysis. The FFR at the LCx was assessed in all patients.

In our study, 447 patients (79.4%) with FFR > 0.8 at LCx after LM‐LAD stenting were considered to have nonsignificant or disease‐free LCx (high‐FFR group) and treated with optimal medical therapy. Alternatively, 116 patients (20.6%) had FFR < 0.8 and were considered to undergo further interventions (low‐FFR group). Three different interventional techniques were used according to the operators preference: kissing balloon inflation (KBI); drug‐eluting balloon (DEB); or conversion to the two‐stent technique. All patients were followed up for a mean of 32.2 ± 8.9 months for major adverse cardiac events (MACE), comprising MI, cardiac and non‐cardiac deaths, revascularizations, and stent thrombosis. A comparison was done between the two FFR groups, as well among the different treatment strategies used for the low‐FFR group. The study was approved by the Xian Jiaotong University Ethical Committee of Medical and Biological Center, Shaanxi, China, and all patients provided informed written consent.

### 2.2 PCI procedure

Angiographic and PCI procedures were performed using trans‐radial or trans‐femoral approaches, and all operations were performed using approved interventional techniques.^10^ All patients received an aspirin loading dose of 300‐mg P2Y12 inhibitors (clopidogrel loading dose: 300–600 mg; ticagrelor loading dose: 180 mg) prior to PCIs. All patients were administered an intravenous bolus of 100‐IU/kg heparin preprocedure, and the dosage was planned according to the activated clotting time maintenance of >250 s throughout the intervention. All patients received drug‐eluting stents (DESs) for LM to LAD by a simple crossover intervention technique, and the stent diameter and length were selected by expert operators according to the reference diameter of the vessels, length of the culprit lesions, plaque burden (PB), and IVUS‐based measurements. Patients in the FFR <0.8 group received further interventions for compromised LCx ostium, with either KBI (40 patients), DEB (37 patients), or stenting (39 patients) based on IVUS and angiographic findings, and the operator's experience. Procedural success for LM to LAD stenting and compromised LCX interventions were defined as lumen stenosis of <25% and with thrombolysis in myocardial infarction (TIMI) flow grade 3 by final coronary angiography. All patients were discharged with standard guidelines and recommended daily loading doses of aspirin (300 mg/day) and clopidogrel (75 mg/day) for the first year postdischarge, and 100‐mg/day aspirin and 75‐mg/day clopidogrel thereafter, in addition to 20‐40‐mg/day atorvastatin and 25.5‐mg/day metoprolol. During follow‐up visits, the medication and dosage continuation or discontinuation were as per patient symptoms, the circumstances of their comorbid diseases, and operator judgment.

### 2.3 IVUS imaging

Pre‐ and post‐PCI IVUS was performed using a commercially available system (Boston Scientific/SCIMED, Minneapolis, MN; or Eagle Eye, Volcano Corporation, Rancho Cordova, CA, USA), and all images were obtained after 0.2‐mg intracoronary nitroglycerine administration. The IVUS catheter was placed >12 mm ahead of the lesion during the pre‐PCI‐IVUS assessments. Prior to balloon inflation, an automatic transducer was pulled back from the LCx to the LM as a side branch (SB), and from the LAD to the LM as the main branch, at a speed of 0.5 mm/s. Two experienced operators assessed and measured pre‐ and post‐PCI MLA, MLD, external elastic membrane (EEM), and PB, which were calculated as follows: EEM‐luminal area/EEM × 100%, for four bifurcation segments using the IVUS offline PC‐software image view and echo plaque (INDEC Systems, Inc., Mountain View, CA, USA).^11^, ^12^

Post‐PCI IVUS with a computerized pull back for stent assessment was repeated for all patients from the LAD to LM and from the LCx to the LM in the stenting subgroup, for stent malposition and stent edge dissection. Minimal stent areas (MSAs) and EEM were measured in the following segments: LAD, POC, LCx, and LMS. Post‐PCI IVUS was repeated to ensure adequate stent expansion, and the ratio of stent expansion was measured as follows: minimum stent cross‐sectional area = (CSA/CSA of proximal reference lumen**+**CSA of distal reference lumen) × 1/2.^13^ Stent expansion was considered to be efficient with a luminal area of 85%–90% or larger than the average preintervention reference luminal area. Patients with stent under‐expansion underwent further non‐compliant balloon postdilation to achieve desirable stent expansion.

### 2.4 FFR measurements

The FFR was measured at jailed LCx with angiographic stenosis >50%, immediately after the LM to LAD crossover stenting, by using a 0.014‐inch pressure guide wire (Radi Medical Systems, Uppsala, Sweden; or Pressure Wire Certus, St. Jude Medical, St. Paul, AK, USA), passing through the distal struts of the stent, and placed 10–12 mm beyond the LCX ostium without any prior balloon inflation at the LCX ostium. The sensor guide wire was positioned at the tip of the guiding catheter to adjust the pressure. FFR measurements were then taken in patients following intracoronary bolus administration of adenosine (150–200 μg) or continuous intravenous (IV) infusion of 120–240‐μg/kg/min adenosine. LCx was considered functionally stenosed in patients with FFR < 0.8.^14^ The IVUS assessment data of the patients with FFR < 0.8 group were compared, and they were further classified into three subgroups according to the additional interventions received: KBI, DEB, and stenting (placement of a second stent).

### 2.5 Definitions and study endpoints

The primary endpoints and clinical outcomes of the current study were the 3‐year MACE incidence. Unless a definite non‐cardiac cause was reported, all deaths were assumed to be cardiac deaths. Myocardial infarction was diagnosed and confirmed by two independent internal adjudicators, based on the development of a new pathological Q‐wave (>0.04 ms) or ST‐elevation (>2 mm) in two contiguous leads on an electrocardiogram (ECG), or greater than the three‐fold elevation of cardiac troponins (T/I) compared to the normal level in more than two samples. Non‐Q‐wave MI was defined as a solely three‐fold increase in cardiac biomarkers (troponins and creatine kinase‐MB (CKMB), with concomitant cardiac chest pain>30 min on average, and absence or partially developed pathological Q‐wave or ST‐elevation changes on ECG.^15^, ^16^ Target lesion revascularization (TLR) was described as a percutaneous revascularization technique involving repetitive stenting, DEB, balloon angioplasty, or surgical bypass grafting for a re‐stenosed or occluded culprit goal lesion within 5 mm of the stent's distal or proximal margins.^10^

In our study, angiographic re‐stenosis was confirmed by two internal expert interventional cardiologists and categorized as diameter stenosis (DS) >50% in a previous LM to LAD stent or in the distal LAD vessel stent, in both FFR > 0.8 and FFR < 0.8 groups; while SB re‐stenosis was diagnosed as angiographic DS > 50% in stented cases, and DS > 65%–70% in the non‐stented SB. Angiographic re‐stenosis for subgroup treatments of the FFR < 0.8 group was defined as DS > 50% for the stenting subgroup, and DS > 50% with concomitant chest pain for DEB and KBI subgroups. Target vessel revascularization, for the FFR > 0.8 group, FFR < 0.8 group, and subgroups, was defined as any repeated PCI or surgical bypass grafting for any de novo stenosis >70% of a coronary artery containing the target lesion. The Academic Research Consortium guidelines were used to identify definite or probable stent thrombosis (ST).^17^ The three‐year composite MACE rate was also compared among the different treatment strategies used for patients with LCx‐FFR < 0.8, after MV crossover stenting.

### 2.6 Statistical analysis

For continuous variables, statistical data were expressed as means and standard deviations, and categorical variables were expressed as numbers and percentages. To compare groups, the independent t‐test or Mann–Whitney U‐test was used for continuous variables, and the exact Chi‐square or Fisher's exact test was used for categorical variables. A multivariable logistic regression model was used to assess the independent predictors of low FFR in the LCx after LM to LAD stenting. Variables that displayed a marginal association on univariable testing (*p* ≤ 0.20), and all IVUS parameters, were entered into the regression model. Sole variables that were significantly correlated with low FFR (*p* < .05) were included in the final regression model. To determine each variable's predictive capacity, the odds ratio (OR) and 95% confidence intervals (CIs) were calculated. The receiver operating characteristic (ROC) curve test was conducted to analyze the optimal cut‐off values for the parameters that independently predicted low FFR poststenting in the LCx. The Kaplan–Meier approach was utilized to calculate the period to the clinical endpoint and survival rate, which were then correlated using the log‐rank test. All *p* values were bipolar. Statistical significance was set at *p* < .05. SPSS v.21 (IBM, Armonk, New York, USA) was used for data management and statistical analysis.

## 3 RESULTS

### 3.1 Patients' clinical and lesion characteristics

An among the 563 patients enrolled, 116 (20.6%) had FFR < 0.8 after crossover stenting (low‐FFR group) and 447 (79.4%) had FFR > 0.8 (high‐FFR group). There were no major variations in the clinical characteristics and risk factors of the sample community between the low and high‐FFR groups (Table 1). The patients' lesion and procedural features are shown in Table 2. Prior to PCI at the lesion level, the low‐FFR group had a smaller minimal lumen region of the LCx but a significantly longer LM‐LAD stent length than the high‐FFR group. After LM‐LAD stenting, patients with FFR < 0.8 showed significantly smaller MLA, MLD of the LCx, and LM with larger PB in both arteries. There were no significant differences in the other IVUS‐measured parameters between the two groups.

**TABLE 1:** Baseline characteristics of the study population

Variables	FFR > 0.8 (*n* = 447)	FFR < 0.8 (*n* = 116)	*p* value
Mean age (years)	62.4 ± 5.2	61.6 ± 5.3	0.121
Females, *n* (%)	139 (31.1)	46 (39.7)	.080
Hypertension, *n* (%)	253 (56.6)	73 (62.9)	0.218
Diabetes, *n* (%)	238 (53.2)	66 (56.9)	0.482
Smoking, *n* (%)	199 (44.5)	49 (42.2)	0.660
Hyperlipidemia, *n* (%)	186 (41.6)	50 (43.1)	0.772
Peripheral arterial Disease, *n* (%)	175 (39.1)	46 (39.7)	0.921
Clinical presentation, *n* (%)			
Chronic stable angina	197 (44.1)	51 (44.0)	0.183
Unstable angina	183 (40.9)	40 (34.5)	
NSTEMI	67 (15.0)	25 (21.6)	
Mean left ventricular ejection fraction (%)	59.6 ± 8.1	59.7 ± 8.2	0.973
Multivessel disease, *n* (%)	213 (47.7)	61 (52.6)	0.343
Moderate/severe calcification, *n* (%)	167 (37.4)	49 (42.2)	0.335
Bifurcation angle	69.8 ± 12.04	69.9 ± 12.07	0.924

*Note*: Data are mean ± *SD*, median (IQR), or number (%) of patients.

Abbreviations: FFR, fractional flow reserve; NSTEMI, non‐ST segment elevation myocardial infarction.

**TABLE 2:** Lesion and procedural characteristics of the study population

Variables	FFR > 0.8 (*n* = 447)	FFR < 0.8 (*n* = 116)	*p* value
Baseline			
LMS			
Minimal lumen area, mm^2^	5.68 ± 0.26	5.66 ± 0.29	0.501
Minimal lumen diameter, mm	1.53 ± 0.08	1.52 ± 0.15	0.778
Plaque burden	69.21 ± 9.89	69.52 ± 7.46	0.624
POC			
Minimal lumen area, mm^2^	5.10 ± 0.11	5.09 ± 0.12	0.852
Minimal lumen diameter, mm	1.52 ± 0.14	1.54 ± 0.17	0.218
Plaque burden	54.68 ± 3.89	54.80 ± 3.77	0.771
LAD			
Minimal lumen area, mm^2^	4.40 ± 0.18	4.41 ± 0.17	0.473
Minimal lumen diameter, mm	1.15 ± 0.17	1.14 ± 0.13	0.427
Plaque burden	71.53 ± 5.75	71.71 ± 6.22	0.759
LCX			
Minimal lumen area, mm^2^	4.89 ± 0.07	4.83 ± 0.19	.002
Minimal lumen diameter, mm	2.25 ± 0.15	2.23 ± 0.14	0.142
Plaque burden	35.45 ± 3.20	35.58 ± 3.09	0.690
LM‐LAD stent			
Diameter (mm)	3.57 ± 0.13	3.55 ± 0.17	0.873
Length (mm)	31.38‐ ± 4.79	32.55 ± 4.45	.017
LM‐LAD lesion length (mm)	25.13 ± 4.81	26.08 ± 4.99	.062
After LM‐LAD stenting			
LMS			
Minimal lumen area, mm^2^	10.56 ± 1.78	10.33 ± 0.53	.024
Minimal lumen diameter, mm	3.52 ± 0.15	3.48 ± 0.16	.015
Plaque burden	16.49 ± 5.54	17.82 ± 6.09	.033
POC			
Minimal lumen area, mm^2^	11.46 ± 0.92	11.41 ± 0.88	0.594
Minimal lumen diameter, mm	3.40 ± 0.056	3.39 ± 0.096	0.537
Plaque burden	17.99 ± 5.38	18.98 ± 5.64	.081
LAD			
Minimal lumen area, mm^2^	9.67 ± 0.38	9.62 ± 0.48	0.286
Minimal lumen diameter, mm	2.78 ± 0.13	2.77 ± 0.25	0.638
Plaque burden	16.01 ± 2.36	16.39 ± 2.78	0.115
LCx			
Minimal lumen area, mm^2^	4.19 ± 0.25	3.84 ± 0.08	<.001
Minimal lumen diameter, mm	2.01 ± 0.17	1.75 ± 0.14	<.001
Plaque burden	52.68 ± 5.32	59.55 ± 2.93	<.001
Mean LCx FFR value	0.87 ± 0.05	0.72 ± 0.04	<.001

*Note*: Data are mean ± *SD* or number (%) of patients.

Abbreviations: FFR, fractional flow reserve; IVUS, intravascular ultrasound; LCx, left circumflex artery; LMS, left main stem; POC, polygon of confluence; LAD, left anterior descending artery.

### 3.2 Clinical follow‐up

**FIGURE 1:**
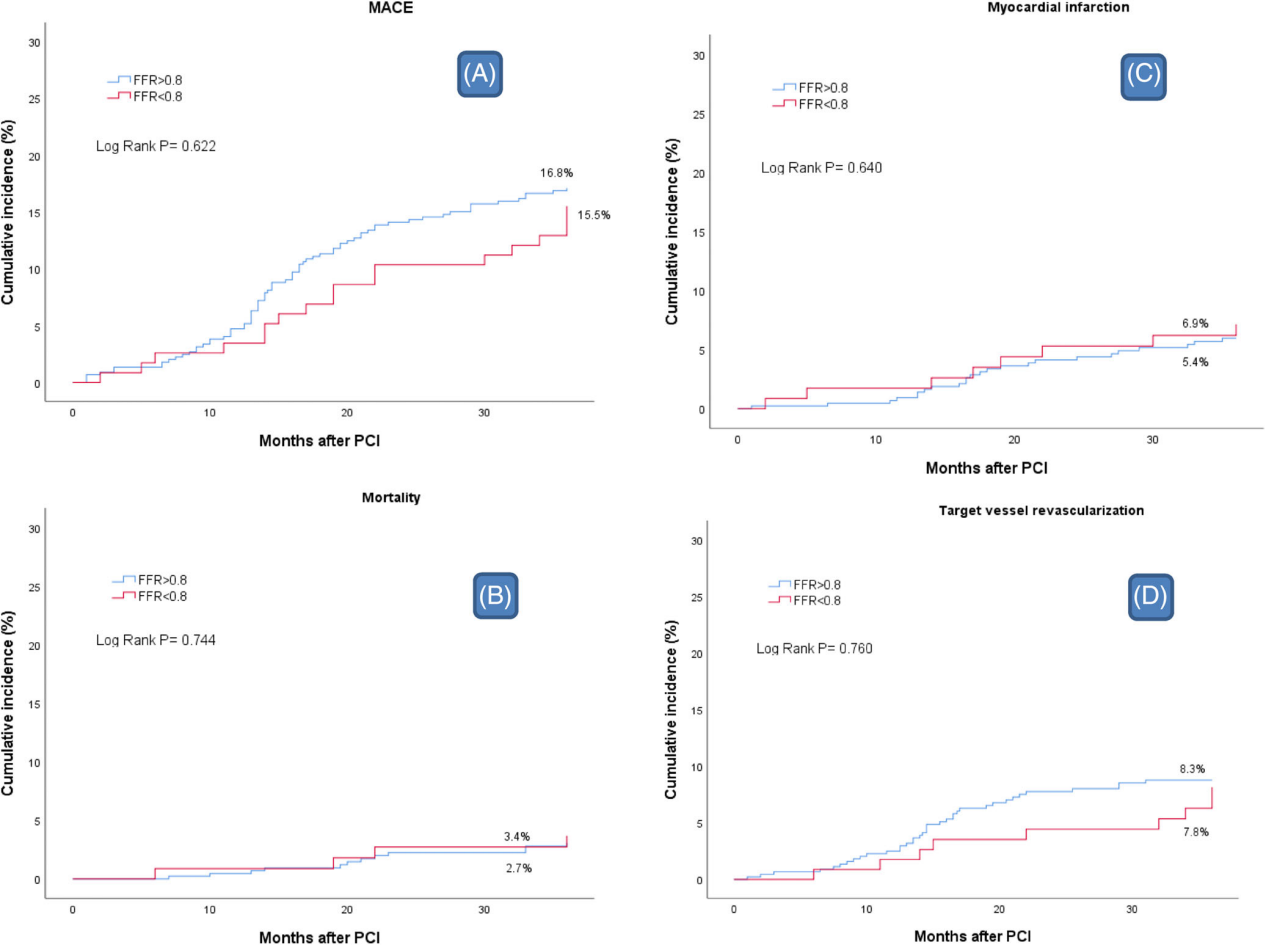
MACE comparison among the groups in three‐years of follow‐up. Cumulative 3‐year event rate according to FFR in LCx. FFR, fraction flow reserve; LCx, left circumflex; MACE, major adverse cardiovascular events (all cause of mortality, reinfarction, ischemia‐driven target vessel revascularization [TVR], or stent thrombosis); PCI, percutaneous coronary interventions

The three‐year clinical outcomes of the two groups are shown in Table 3 and Figure 1. The three‐year composite MACE rates were 16.8% and 15.5% in the high‐ and low‐FFR groups, respectively (*p* = 0.744). The need for revascularization during the follow‐up period was the most common event in both the high‐ and low‐FFR groups (8.3% vs. 7.8%, respectively; *p* = 0.856). The mortality rate was not significantly different between the two groups (2.7% vs. 3.4%; *p* = 0.659). The myocardial infarction (MI) rate was diagnosed in 5.4% and 6.9% for the high‐ and low‐FFR groups, respectively (*p* = 0.527). Definite/probable ST occurred in 1.1% and 2.6% of patients in the high‐and low‐FFR groups, respectively, which required further (SB) intervention (*p* = 0.234).

**TABLE 3:** 3‐year clinical outcomes of the overall study population and according to different treatment strategy of the compromised left circumflex artery

Variables	Total(*n* = 563)	FFR > 0.8(*n* = 447)	FFR < 0.8(*n* = 116)	*p* value	DEB (*n* = 37)	KBI(*n* = 40)	Stenting(*n* = 39)	*p* value
MACE, *n* (%)	93 (16.5)	75 (16.8)	18 (15.5)	0.744	3 (8.1)	7 (17.5)	8 (20.5)	0.299
Mortality (all), *n* (%)	16 (2.8)	12 (2.7)	4 (3.4)	0.659	0 (0.0)	3 (7.5)	1 (2.6)	0.184
Cardiac	9 (1.6)	6 (1.3)	3 (2.6)	0.341	0 (0.0)	2 (5.0)	1 (2.6)	0.385
Myocardial infarction, *n* (%)	32 (5.7)	24 (5.4)	8 (6.9)	0.527	2 (5.4)	2 (5.0)	4 (10.3)	0.595
Q‐wave	10 (1.8)	6 (1.3)	4 (3.4)	0.126	1 (2.7)	1 (2.5)	2 (5.1)	0.779
Revascularization, *n* (%)	46 (8.2)	37 (8.3)	9 (7.8)	0.856	1 (2.7)	4 (10.0)	4 (10.3)	0.379
TLR	34 (6.0)	27 (6.0)	7 (6.0)	0.998	0 (0.0)	4 (10.0)	3 (7.7)	0.159
Definite or probable stent thrombosis, *n* (%)	8 (1.4)	5 (1.1)	3 (2.6)	0.234	0 (0.0)	1 (2.5)	2 (5.1)	0.371

*Note*: Data are number (%) of patients.

Abbreviations: DEB, drug‐eluting balloon; KBI, kissing balloon inflation; MACE, major adverse cardiovascular events (all‐cause of mortality, reinfarction, ischemia‐driven target vessel revascularization, or stent thrombosis).

Regarding the intervention strategy in SB, patients with FRR < 0.8 in the LCx after MV stenting were managed with either stent deployment in LCx (39 patients), KBI alone (40 patients), or DEB (37 patients). The overall pooled three‐year MACE rates tended to be lower in the DEB group than in the KBI or stenting subgroup (8.1% vs. 17.5% vs. 20.5%), but this tendency did not reach statistical significance (*p* = 0.299) (Table 3).

### 3.3 Predictors for low FFR

Multivariable stepwise regression analysis identified poststenting MLA of the LCx (OR: 0.032; 95% CI: 0.011–0.052, *p* < .001), poststenting LCx PB (OR: 1.166; 95% CI: 1.104–1.232, *p* < .001), poststenting LM MLA (OR: 0.821; 95% CI: 0.681–0.989, *p* = .038), and prestenting LCx MLA (OR: 0.371; 95% CI: 0.141–0.975; *p* = .044) as significant independent predictors of low FFR in the LCx (Table 4). Poststenting MLA within the LCx ostium of <3.9 mm^2^ predicted a poststenting FFR of <0.80, with a sensitivity of 88.6% and specificity of 85.5% (area under the curve [AUC] = 0.885, 95% CI 0.852–0.918, *p* < .001). A poststenting PB at the LCx of >55.6% predicted a poststenting FFR < 0.80, with a sensitivity of 84.4% and a specificity of 83.8% (AUC: 0.856; 95% CI: 0.822–0.891, *p* < .001); however, for poststenting LM MLA and prestenting LCx MLA, there were no cut‐off values showing a significant correlation with poststenting low FFR on ROC curve analysis.

**TABLE 4:** Multivariable regression analysis of predictors for low FFR

Variables	OR	95% CI for OR	*p* value
Poststenting LCx MLA, mm^2^	0.032	0.011–0.052	<.001
Poststenting LCx plaque burden %	1.166	1.104–1.232	<.001
Poststenting LM MLA, mm^2^	0.821	0.681–0.989	.038
Prestenting LCx MLA, mm^2^	0.371	0.141–0.975	.044

Abbreviations: 95% CI, 95% confidence interval; LCx, left circumflex; LM, left main; PCI, percutaneous coronary intervention; MLA, minimal lumen area; OR, odds ratio.

## 4 DISCUSSION

The results of the current study consist of three core findings: (1) the importance of FFR‐guided intervention in avoiding unnecessary procedures for apparently significant jailed LCx lesions after LM to LAD stenting; (2) the identification of IVUS predictors of patients with FFR < 0.8, after MV stenting; and (3) the comparison of different management strategies for patients requiring LCx intervention. SB FFR and IVUS assessments were performed in all 563 patients. Only 116 patients (20.6%) showed a significant decrement in FFR < 0.8 after MV stenting, which was associated with IVUS‐measured post‐PCI MLA of LCx (cut‐off value of 3.9 mm^2^ predicted FFR <0.80, with 88.6% sensitivity and 85.5% specificity; *p* < .001) and LCx PB (cut‐off value of 55.6% predicted FFR <0.8, with 84.4% sensitivity and 83.8% specificity; *p* < .001) in addition to poststenting LM MLA and prestenting LCx MLA.

The three‐year clinical outcomes, in terms of composite MACE, among the two groups (FFR > 0.8 and FFR < 0.8) after LM to LAD stenting were identical (16.8% vs. 15.5%; *p* = 0.744). In patients requiring further intervention guided by post‐PCI with significantly low FFR, there was a trend toward decreased three‐year MACE rate and improved clinical outcomes in the DEB group, compared to the stenting and KBI subgroups; however, this trend was not statistically significant (MACE rates: 8.1%, DEB subgroup vs. 20.5%, stenting subgroup and 17.5%, KBI subgroup, *p* < 0.299).

The SB flow was acceptable (TIMI flow 3) in 79.4% of the patients who had undergone a single‐stent strategy (from LAD to LM) of the bifurcation lesion and achieved postprocedural IVUS, LCx MLA of 4.19 ± 0.25 mm^2^, LCx‐MLD of 2.01 ± 0.17 mm, and PB of 52.68% ± 5.32%. However, only 116 (20.6%) patients showed a functionally significant FFR of <0.8. The main mechanisms for SB compromise include carina and plaque shift, in addition to SB vasospasm and presence of metal struts of the deployed MV stent.^18^, ^19^ In the current study, the independent predictors of compromised flow in the LCx were lower poststenting MLA within the LM and LCx ostium, higher poststenting PB at the LCx, and lower prestenting LCx MLA.

Kang et al. showed similar predictive factors for compromised LCx after LM to LAD stenting, with a fall of pre‐to‐post‐IVUS LCx‐MLD and MLA; although it was also signified that, within the LCx ostium, limited IVUS‐MLA could often not indicate functionally relevant stenosis.^20^ In their research, postprocedural MLA within the <3.7 mm^2^ LCx ostium anticipated a poststenting FFR of <0.80, with 100% sensitivity and 71% specificity, although the PB shift from the MV to the ostium after MV stenting has positive significance with the FFR decrement at the LCx ostium.^20^, ^21^ Oviedo et al. also showed that 29% of patients have carina shift according to the conclusion of LCx‐angiographic DS >50% after LM to LAD stenting, with a functionally significant FFR <0.8,^22^, ^23^ while Koo et al. reported that only 27% of patients with angiographic stenosis >75% of the SB had an FFR <0.8, and SB with angiographic stenosis <75% had no functionally significant FFR.^9^

Additionally, increased stent length was used to cover diffuse atherosclerotic disease, which contains a large PB, and the associated pre and postdilation will cause increased plaque shift to LCx and more flow reduction.^24^, ^25^ An increased stent**/**artery ratio was also found to accentuate plaque shifting to the SB.^26^ In the current study, increased total deployed MV stent length was significantly associated with decreased poststenting FFR in LCx in the univariate analysis, but it did not appear to be an independent predictor in the regression analysis.

The three‐year clinical outcomes were compared as a composite MACE, and we found no statistically significant difference between the high and low FFR groups (16.8% vs. 15.5%; respectively; *p* = 0.744). Although the incidence rates of revascularization were higher in the FFR > 0.8 group (8.3% vs. 7.8%), and the incidence rates of Myocardial infarction MI and stent thrombosis ST were higher in the FFR <0.8 group (5.4% vs. 6.9% and 1.1% vs. 2.6%), the difference was not statistically significant (*p* > .05).

Contrarily, Lee et al. presumed that the high‐FFR group had better 5‐year outcomes than the low‐FFR group, with disclosure of improvised TLR in the low‐FFR group at LCx, due to proliferation of plaque at the LCx ostium.^4^ Additionally, Cho et al. showed a similar MACE rate of 18.1% at 1 year in an FFR and angiographic‐guided randomized study, with a slightly higher incidence of revascularization in the main and side branch vessels in the angiographic‐guided PCI, and suggested that IVUS may offer sensitive details regarding the anatomical alteration associated with a bifurcation lesion.^27^, ^28^ Furthermore, Karrowni et al. found in their observational study that a single‐stent strategy in LM stem bifurcation‐lesion with mild ostial LCx disease has lower MACE and revascularization rates at 32 months of follow‐up.^29^

There have been few studies on additional LCx‐PCI deferred based on FFR. The current study is the first to compare DEB, stenting, and KBI in low FFR patients after LM‐LAD stenting. We found that DEB was associated with better clinical outcomes, whereas placing another stent and simple performing KBI showed comparatively higher MACE rates and increased the need for future revascularization. The three‐year composite MACE was compared among the three subgroups: 8.1% in patients treated with DEB, 17.5% in patients who underwent KBI only, and 20.5% in patients who received another stent (*p* = 0.299). In the clinical context of 37 patients receiving DEB, there was no mortality, while only two cases of myocardial infarctions and only one case required repeated revascularization at the three‐year follow‐up.

The present study highlighted the relatively higher incidence of MACE and MIs in the stenting SB subgroup, and a higher incidence of deaths in the KBI subgroup than in the DEB subgroup, which could be related to positive plaque and carina shift with remodeling of the LCx ostium after LM stenting and the absence of actual significant atherosclerotic disease in the LCx. However, this trend did not reach statistical significance, and further studies with larger populations are necessary to confirm the long‐term benefits of DEB in comparison to new‐generation stents in this setting.^30^ In parallel with our results, Jeger et al. demonstrated the same benefits of DEB versus DES for small coronary artery disease PCI in an open‐label randomized non‐inferiority trial.^31^ This finding is supported by recent reports emphasizing DEB is a promising technique for certain de novo coronary lesions, with improved safety and efficacy.^32^ According to Hirohata et al., directional atherectomy in conjunction with DEB improves outcomes in LCx ostial stenosis and could soon be considered as a stentless therapeutic option.^33^

Conversely, Koo et al. showed that stenting had better clinical outcomes in patients undergoing SB‐PCI with low FFR after LM compared to LAD stenting.^21^ Similarly, Tanaka et al. showed in their observational study that lesions deferred had FFR < 0.8, and patients with dyslipidemia had a higher incidence of MACE and myocardial infarction (*p* = .003) compared to deferred patients with FFR > 0.8.^34^

## 5 STUDY LIMITATIONS

This analysis had numerous limitations. First, this study was a retrospective analysis of a single core. Second, patients classified as (1.1.1) by Medina, patients with acute myocardial infarction, and patients with previous PCI or CABG as an index procedure to treat, were excluded from the study, resulting in many unprotected LMS lesions. Limited pre‐ and post‐IVUS parameters regarding vessel size and lesion assessment were analyzed, and the stent and balloon diameters, lengths, and MSA of the subgroups of patients with low FFR were not included in the final analysis, which could have provided additional information. Third, the current study used a cut‐off value of 0.80 for FFR‐directed interventions, despite the fact that the exact cut‐off value for FFR is still controversial (0.75–0.8); most interventional cardiologists use 0.8, which may have influenced the final results. Finally, the cases available for subgroup analysis in patients with low FFR after MV stenting were limited.

## 6 CONCLUSION

Most of the complexity in the management of distal LM lesions is regarding the side branch. FFR‐guided LCx interventions may be useful in avoiding unnecessary complex interventions. Poststenting MLA and PB of the LCx, as well as poststenting MLA of the LM and prestenting MLA of the LCx, are IVUS‐guided predictors of low FFR after MV stenting. The three‐year MACE rates were comparable between patients with high FFR in LCx and patients with low FFR who were managed with DEB, KBI, or another stent deployment. DEB treatment of compromised LCx resulted in the fewest three‐year clinical adverse events.

## CONFLICT OF INTEREST

The authors declare no conflicts of interest.

## Supporting information


**Appendix S1**: Supporting Information

**Appendix S2**: Supporting Information

## Data Availability

The data used to support the findings of this study are available from the First Affiliated Hospital of Xi'an Jiaotong University. The data are available from the corresponding authors upon reasonable request and with permission from the hospital.
