# Supplementary material for: Predictors and 3‐year outcomes of compromised left circumflex coronary artery after left main crossover stenting
Source: Clin Cardiol. 2021 Jul 16;44(10):1377–85. doi: 10.1002/clc.23693 (PMC8495093; doi:10.1002/clc.23693)
Supplement: Supplementary file 1 — Appendix S1: Supporting Information [file CLC-44-1377-s001.docx]

**Flowchart of the present study population.**

**(n=1005) patients after LM-LAD stenting have LCx angiographic stenosis < 50%**

**Total n= 563 patients received the LCx-FFR immediately after LM-LAD stenting**

**Total n= 577 patients received the LCX-FFR immediately after LM-LAD stenting, except 14 patients who failed FFR, LCx-Rewiring**

**(n=577) patients after LM-LAD stenting have LCx angiographic stenosis > 50%**

**LM: left main coronary artery, IVUS: intra vascular ultrasound, FFR: Fractional flow reserve, LAD: Left anterior descending, LCx: Left circumflex, MACE: major adverse cardiac events, LVEF: Left ventricular ejection fraction, PCI: Percutaneous coronary interventions, DEB: Drug eluting balloon, KBI: Kissing balloon inflation.**
