# Supplementary material for: Predictors and 3‐year outcomes of compromised left circumflex coronary artery after left main crossover stenting
Source: Clin Cardiol. 2021 Jul 16;44(10):1377–85. doi: 10.1002/clc.23693 (PMC8495093; doi:10.1002/clc.23693)
Supplement: Supplementary file 2 — Appendix S2: Supporting Information [file CLC-44-1377-s002.docx]

**ROC curves**

**1. Post-PCI LCX-MLA**


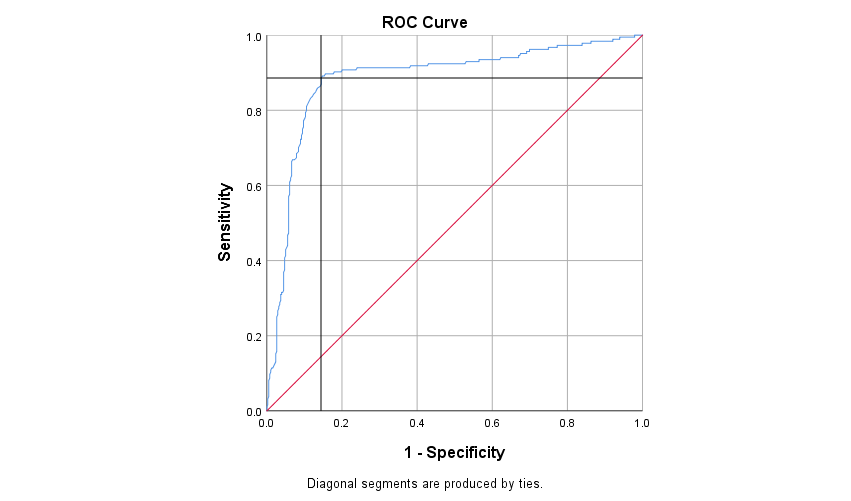


| **Area Under the Curve** | | | | |
| --- | --- | --- | --- | --- |
| Test Result Variable(s) | | | | |
| Area | Std. Error^a^ | Asymptotic Sig.^b^ | Asymptotic 95% Confidence Interval | |
|  |  |  | Lower Bound | Upper Bound |
| .885 | .017 | .000 | .852 | .918 |

**Cut value 3.95 mm**

**Sensitivity: 88.6%**

**Specificity: 85.5%**

**2. Post-PCI Plaque Burden of LCX**


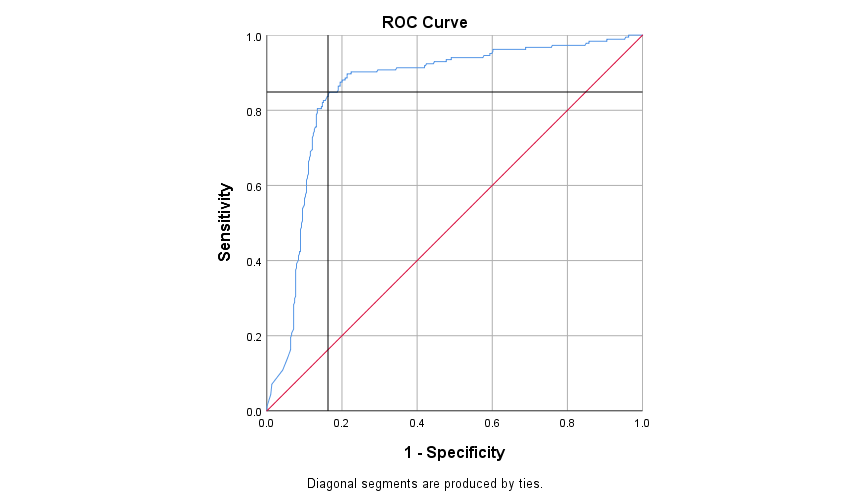


| **Area Under the Curve** | | | | |
| --- | --- | --- | --- | --- |
| Test Result Variable(s) | | | | |
| Area | Std. Error^a^ | Asymptotic Sig.^b^ | Asymptotic 95% Confidence Interval | |
|  |  |  | Lower Bound | Upper Bound |
| .856 | .018 | .000 | .822 | .891 |

**Cut value :55.6**

**Sensitivity: 84.8%**

**Specificity: 83.8%**
